# Electronic and Optoelectronic Monolayer WSe_2_ Devices via Transfer-Free Fabrication Method

**DOI:** 10.3390/nano13081368

**Published:** 2023-04-14

**Authors:** Zixuan Wang, Yecheng Nie, Haohui Ou, Dao Chen, Yingqian Cen, Jidong Liu, Di Wu, Guo Hong, Benxuan Li, Guichuan Xing, Wenjing Zhang

**Affiliations:** 1 Joint Key Laboratory of the Ministry of Education, Institute of Applied Physics and Materials Engineering, University of Macau, Avenida da Universidade, Taipa, Macao SAR 999078, China; 2 International Collaborative Laboratory of 2D Materials for Optoelectronics Science and Technology of Ministry of Education, Institute of Microscale Optoelectronics, Shenzhen University, Shenzhen 518060, China; 3 Department of Materials Science and Engineering & Center of Super-Diamond and Advanced Films, College of Engineering, City University of Hong Kong, 83 Tat Chee Avenue, Kowloon, Hong Kong SAR 999077, China; 4 Electrical Engineering Division, Engineering Department, University of Cambridge, 9 JJ Thomson Avenue, Cambridge CB3 0FA, UK

**Keywords:** chemical vapor deposition, monolayer WSe_2_, transfer-free, transistor, photodetector

## Abstract

Monolayer transition metal dichalcogenides (TMDs) have drawn significant attention for their potential applications in electronics and optoelectronics. To achieve consistent electronic properties and high device yield, uniform large monolayer crystals are crucial. In this report, we describe the growth of high-quality and uniform monolayer WSe_2_ film using chemical vapor deposition on polycrystalline Au substrates. This method allows for the fabrication of continuous large-area WSe_2_ film with large-size domains. Additionally, a novel transfer-free method is used to fabricate field-effect transistors (FETs) based on the as-grown WSe_2_. The exceptional metal/semiconductor interfaces achieved through this fabrication method result in monolayer WSe_2_ FETs with extraordinary electrical performance comparable to those with thermal deposition electrodes, with a high mobility of up to ≈62.95 cm^2^ V^−1^ s^−1^ at room temperature. In addition, the as-fabricated transfer-free devices can maintain their original performance after weeks without obvious device decay. The transfer-free WSe_2_-based photodetectors exhibit prominent photoresponse with a high photoresponsivity of ~1.7 × 10^4^ A W^−1^ at V_ds_ = 1 V and V_g_ = −60 V and a maximum detectivity value of ~1.2 × 10^13^ Jones. Our study presents a robust pathway for the growth of high-quality monolayer TMDs thin films and large-scale device fabrication.

## 1. Introduction

The emergence of two-dimensional (2D) semiconductors, specifically transition metal dichalcogenides (TMDs), has garnered significant interest in high-performance electronic devices due to their unique transition from indirect to direct band gaps when reducing the number of layers to one [1,2,3]. For example, bulk WSe_2_ is a p-type semiconductor with an indirect band gap of ~1.2 eV, while monolayer WSe_2_ has a direct band gap of ~1.65 eV, making it suitable for its optoelectronic applications [4]. Though exfoliated WSe_2_ has been used to fabricate high-performance p-type field-effect transistors (FETs) [5], the mechanical exfoliation method limits the size of monolayer semiconductors to a few tens of micrometers. Compared to mechanical cleavage and liquid exfoliation, chemical vapor deposition (CVD) is a well-developed method for growing large-area monolayer TMDs on various substrates, which provides a great potential to obtain van der Waals (vdW) contacts with metals such as graphene grown on Cu (111) film [6] and hBN grown on Cu (110) film [7]. Gao and co-workers demonstrated an ultrafast growth method for producing high-quality WSe_2_ with millimeter-size single-crystal domains on Au foils [8]. Another study showed that researchers can grow single-crystal TMD films on a centimeter scale using the atomic sawtooth gold surface [9]. Furthermore, it has been reported that Au can form vdW interfaces with TMDs due to its good chemical stability in a chalcogen-rich environment [10]. However, the aforementioned monolayer WSe_2_ needs to be transferred onto the dielectric layer for device fabrication, which rises significant issues with cracks, wrinkles, interfacial contamination and transfer size limitation [11,12]. Furthermore, the removal of supporting polymer films, such as poly (methyl methacrylate) (PMMA) usually requires aggressive chemical treatments, resulting in unavoidable contamination on 2D material surfaces and undesirable device performance [13].

Here, we present a large-scale CVD method for growing monolayer WSe_2_ on Au foil with an area of up to 1 cm^2^. We also demonstrate a transfer-free fabrication method for field-effect transistors (FETs) using this monolayer thin film. The spectroscopic and microscopic studies reveal the excellent crystalline quality of the atomically thin WSe_2_. By applying a novel transfer-free fabrication method, vdW integration of Au electrode remains, further FIB-STEM revealing the fine contact and clean interface between metals and 2D semiconductors. Based on this, we observed a robust and consistent p-type characteristic in monolayer WSe_2_, and electrical transport studies further demonstrate that the p-type WSe_2_ FETs exhibit excellent electronic characteristics compared to those with evaporated Au electrodes. Optoelectronic characterizations also show prominent photoresponse, with high photoresponsivity and detectivity in photodetectors fabricated by our novel transfer-free method.

## 2. Experimental Section

### 2.1. Au Foil Preparation

The preparation of polycrystalline Au foils follows our previously reported process [14]. To start, commercially available Au foils (Alfa Aesar, Tewksbury, MA, USA, 25 μm thickness, 99.985% metal basis, LOT: R23F014) were cut into an appropriate size (≈1 cm^2^). Then, small pieces of Au foils were ultrasonically cleaned with an acetone solution and Isopropyl alcohol (IPA) solution for 10 min, respectively. Afterwards, the cleaned Au foils were annealed in the CVD furnace (Kejing, Hefei, China) at 1000 °C for 3 h to release the stress and expose the grain boundaries. Ar with a flow rate of 100 sccm was kept throughout the whole annealing process.

### 2.2. CVD Growth Process of Monolayer WSe_2_

The as-prepared polycrystalline Au foil was placed on a self-designed flattened quartz plate and surrounded by WO_3_ powder (Sigma, St. Louis, MO, USA) (99.9%, 10 mg) (Figure S1a). The plate was then loaded onto a quartz boat and placed at the centre of the heating zone. Selenium powder (99.9%, 20 mg) (Alfa Aesar, Shanghai, China) was put into another boat upstream outside the furnace. The temperature was raised to 900 °C and kept at 900 °C for 10 min to initiate the growth of the WSe_2_ monolayer on Au foil. At last, the furnace was cooled down to room temperature slowly. The H_2_ flow rate was only supplied with 4 sccm during the 10 min growth process, and Ar was used as the carrier and protective gas with a flow rate of 80 sccm throughout the process.

### 2.3. Transfer WSe_2_ Film to SiO_2_/Si Substrate

Poly methyl methacrylate (PMMA) (Aladdin, Shanghai, China) (10 wt.%, in anisole) was spin-coated at 2000 rpm for 60 s and then was baked at 180 °C for 5 min. The PMMA–WSe_2_–Au foil was then placed in the Au etchant solution (I_2_ and KI in a mol ratio of 1:1, dissolved in 50 mL deionized (DI) water) at 50 °C for 1 h to ensure the complete removal of Au foil. After that, the floating film was transferred into DI water to remove the etchant ions and was finally lifted onto a cleaned SiO_2_/Si substrate. The substrate was then dipped into acetone to remove the PMMA layer.

### 2.4. Device Fabrication

After the CVD growth process, 5% PMMA (350-k PMMA dissolved in ethyl lactate) was first coated onto WSe_2_ film using spin-coating at 2500 rpm for 40 s and then baked at 180 °C for 5 min. The photoresist was spin-coated onto Au foil on the side without monolayer WSe_2_ film at 3500 rpm for 40 s and baked at 130° for 3 min. A pattern, as shown in Figure S1b, was designed by CleWin 5.0 (2019, version5.0, WieWeb software, Hengelo, The Netherlands) with a 100 μm channel (exposed part) and 450 μm electrode width (remaining part). The sample was exposed using a laser writer (MicroWriter ML 3, Durham Magneto Optics Ltd., London, UK). The exposed photoresist was developed by NMD-3 (Tokyo Ohka Kogyo Co., Ltd., Tokyo, Japan) for 30 s and rinsed in DI water for another 30 s. After that, the ethyl lactate solution was spin-coated at 1000 rpm for 30 s to form a uniform solution film on the surface of the SiO_2_/Si substrate, followed by rapidly attaching it to the PMMA side and baking at 120 °C for 30 min to obtain the sandwich structure. Next, the Au etchant solution was used to etch away the exposed part of Au by floating the substrate on the Au etchant, resulting in a specific-length WSe_2_ channel. Finally, the overlying photoresist on Au was removed by N-methyl-2-pyrrolidone (NMP) vapor treatment. The comparison devices were fabricated using WSe_2_ CVD-grown on SiO_2_/Si, with 100 nm Au electrodes deposited using masks via the thermal deposition system (PRO Line PVD 75, Shanghai, China).

### 2.5. Characterization

The optical images were obtained using a microscope (Leica DM2700M RL, Wetzlar, Germany). Atomic force microscopy characterization was performed on a Bruker Dimension ICON microscope (365 Boston Rd., Billerica, MA, USA). Raman and Photoluminescence spectra were collected using a Raman spectroscope (Alpha 300, WITec with 532 nm laser, Lise-Meitner-Str., 6 D-89081 Ulm, Germany) and Transmission Electron Microscope images were analyzed by TEM (JEM-3200FS, JEOL, Street No. 6, Haidian District, Beijing, China). The electrical and optoelectronic characteristics of devices were measured using a Keithley 4200 semiconductor characterization system connected to a Lakeshore cryogenic probe station under vacuum conditions. A supercontinuum spectrum white light laser (SC400-8, Fianium Ltd., Southampton, UK) was used as the light source coupled with a monochromator. The light intensity was studied with a Thorlabs commercial power meter.

## 3. Results and Discussion

### 3.1. Synthesis and Characterization of Monolayer WSe_2_

**Figure 1:**
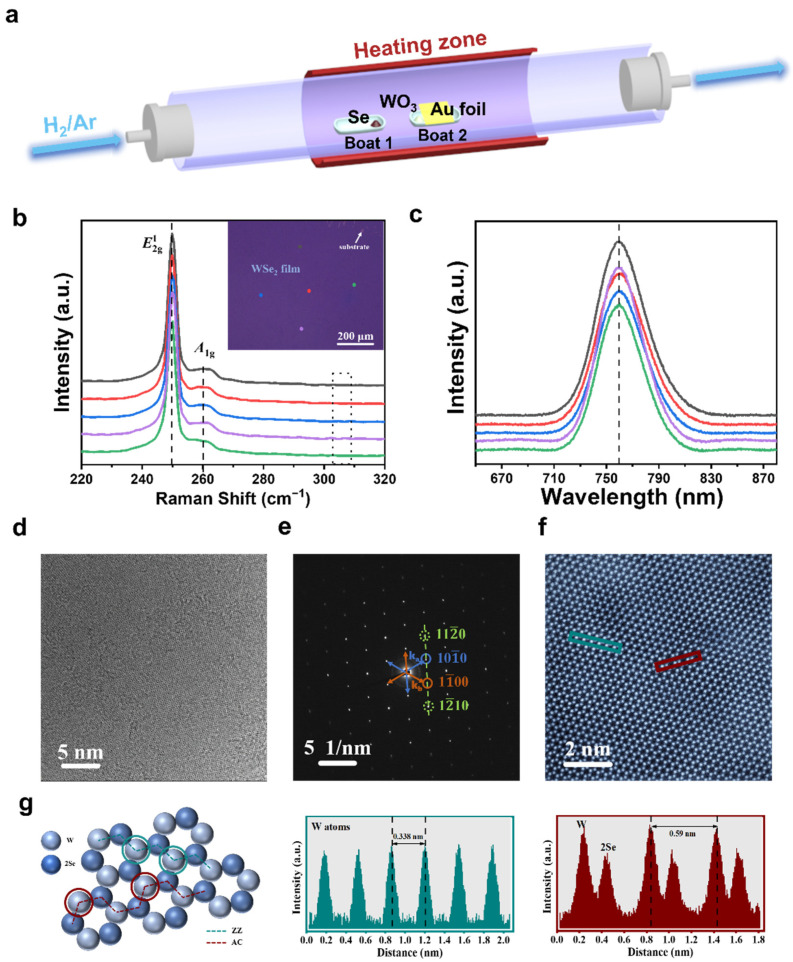
(**a**) Schematics of the CVD setup for the growth of monolayer WSe_2_ film on the polycrystalline Au foil. Raman spectra (**b**) and PL spectra (**c**) were collected from five random points of the WSe_2_ sample marked in the insert pictures. (**d**) Low-magnification TEM image of the WSe_2_ film. (**e**) Typical SAED pattern of the sample in (**d**). (**f**) The colored STEM image of monolayer WSe_2_ film. (**g**) Atomic structure mode of monolayer WSe_2_ film. Corresponding intensity profiles along the zigzag direction (green) and the armchair direction (red) are labelled in (**f**), respectively.

A monolayer WSe_2_ film was fabricated using a one-pot CVD process at atmospheric pressure. The synthesis of the monolayer WSe_2_ film on Au foil is described in the Materials and Methods section in detail. The experiment setup is illustrated in Figure 1a. Note that the Au foil was placed on a specially designed flattened quartz boat, surrounded by WO_3_ powder, which ensures sufficient supplies and uniform distribution of precursors to form large-scale WSe_2_ film on polycrystalline Au substrate. The 10 min stage and the slow cooling stage were applied to grow wrinkle-free monolayer WSe_2_ film. The temperature program used is illustrated in Figure S2a. As shown in Figure S2b, as-grown monolayer WSe_2_ film on Au foil is continuous and without cracks over a large area.

To confirm the uniformity of the as-grown WSe_2_ film, spectroscopic analyses are carried out. Given that the laser exciton would lead to an energy transfer from the WSe_2_ to Au, the as-grown WSe_2_ film was transferred onto SiO_2_/Si substrates to accurately characterize the WSe_2_ structures [8]. The typical Raman spectra (Figure 1b) are obtained from five random positions, as labelled in the optical image shown in the inset. Two obvious characteristic peaks are observed in the region from 249.8 cm^−1^ to 259.6 cm^−1^, which can be assigned to $E_{2g}^{1}$ (in plane) and A_1g_ (out of plane) modes of 2H-WSe_2_, respectively. Meanwhile, the absence of the B_2g_ peak (which is a fingerprint of few-layer WSe_2_ and is absent in monolayer WSe_2_) at ~304 cm^–1^ suggests that the as-grown WSe_2_ film is monolayer [15]. In addition, Raman curves are identical to each other, without any detectable difference in peak frequencies, negligible variations in peak intensities and full-width at half-maximum of each peak, which further confirms the consistently good quality over the whole WSe_2_ film [16]. Moreover, the PL spectra in Figure 1c acquired randomly from five positions also show the same sharp peak at ≈758 nm with a full width at half-maximum of ≈39 nm, a characteristic of direct band gap semiconductors [17]. Figure S3 shows the high-magnification optical microscopic image of the as-grown WSe_2_ film on SiO_2_/Si substrate, indicating its uniform optical contrast and lack of adlayers. The few folded areas at the edge of the WSe_2_ film are due to the transfer process. AFM was applied to further characterize the thickness and morphology properties of the as-prepared WSe_2_ film. The surface quality of the film is evaluated by calculating the root means square roughness (R_q_) over the area shown in Figure S2c, which is 0.425 nm, similar to previously reported values [18]. Figure S2d presents the AFM image of the edge on the WSe_2_ film and the corresponding height profile acquired along the yellow section dash line. The inset curve displays a real film thickness of ≈0.9 nm, consistent with the value in the literature [16]. All these results indicate that the as-grown WSe_2_ film on Au foil is a uniform monolayer.

To determine the quality of as-growth WSe_2_ film, transmission electron microscopy (TEM) and atomic resolution spherical aberration-corrected transmission electron microscopy (ARSTEM) were conducted on the transferred samples. Figure 1d shows a TEM image, indicating that the as-grown monolayer WSe_2_ film has clear crystalline without obvious defects over a wide dimension range. The corresponding selected area electron diffraction (SAED) pattern is provided in Figure 1e, with two different sets of spots. Additionally, ARSTEM (as shown in Figure 1f) reveals its defect-free atomic lattice, and the two sets of spots k_a_ and k_b_ are broken from the asymmetry of W and Se sublattices in monolayer WSe_2_. These are k_a_ = {(1100), (1010), (0110)} and k_b_ = −k_a_ marked with blue and orange color rectangles, respectively. The contrast analysis of the diffraction spots (Figure S4) shows that the k_a_ spots are ~8% brighter than the k_b_ spots, which confirms WSe_2_ is a hexagonal lattice structure with threefold symmetry. Figure 1g is an atomic structure model of monolayer WSe_2_ corresponding to STEM results. Two different orientations determine the distance between two adjacent W atoms: one is the zigzag direction (ZZ) and the other is the armchair direction (AC), highlighted with green and red dash lines, respectively. In theory, the ratio of the distance along AC (D_ac_) and the distance along the ZZ (D_zz_) should be $\sqrt{3}$. Since the intensity of atomic brightness is strongly dependent on the atomic number with an exponential relationship, two random zooms that contain W atoms were selected to study the intensity evolution in the defined direction ZZ and AC with the green and red rectangles marked in Figure 1f. It is clear that the D_zz_ = 0.338 nm and D_ac_ = 0.585 nm for two adjacent W atoms along two labelled orientations, respectively. The experimental ratio of D_ac_ and D_zz_ is about 1.73 and the result is in line with the theoretical values mentioned above, proving that as-grown monolayer WSe_2_ film exhibits very high crystalline quality.

### 3.2. Design of the Transfer-Free Monolayer WSe_2_-Based Device

Since Au is a very good conductor, a novel transfer-free technology route is demonstrated, where the field effect transistors are directly fabricated without separating CVD-grown TMD materials and metal substrate. Our result shows that Au foil could be used as electrodes (source and drain) for field-effect transistors.

**Figure 2:**
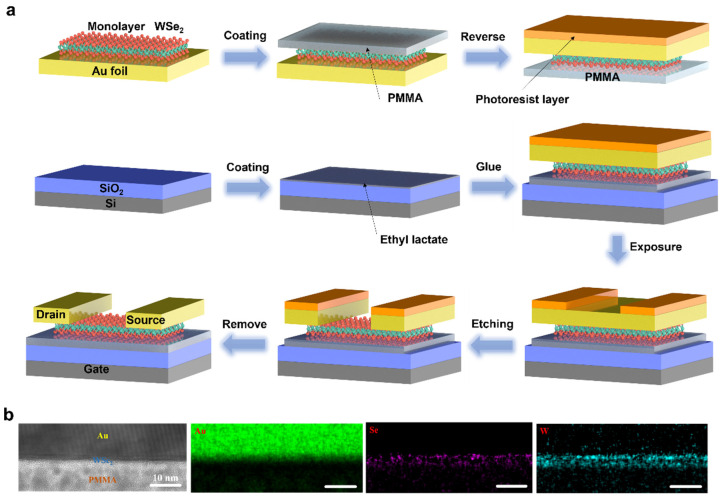
(**a**) Schematic illustration of the transfer-free fabrication process of monolayer WSe_2_ FETs. (**b**) HRTEM cross-sectional images of an as-grown WSe_2_ on the Au foil, with element distribution pictures. Scale bar is 10 nm.

The schematic illustration of the transfer-free FET device fabrication process is presented in Figure 2a. The detailed parameter is described in the experimental section. The uniform monolayer WSe_2_ film on Au foil is used directly after CVD growth without further post-treatment. First, PMMA layer as a supporting scaffold is spin-coated on the WSe_2_ surface to protect the material before the subsequent process. Then, the as-processed flake is reversed, with the blank Au foil side facing up and coated with the photoresist. Thus, a sandwich structure is constructed, composed of a photoresist layer, Au foil, monolayer WSe_2_ film and PMMA layer. The top view pictures of the structure corresponding to the different stages are labelled with different colored rectangle marks, as shown in Figure S5. The ethyl lactate solution is then spin-coated onto a heavily doped silicon substrate with 300 nm silicon oxide, which rapidly sticks with the PMMA side on the as-processed sandwich component. After that, a photoresist layer is patterned and exposed via laser writer, followed by the application of Au etchant solution to etch away the exposed part of Au and form the device electrodes. Finally, the photoresist on Au electrodes is removed by N-methyl-2-pyrrolidone (NMP) vapor and the FET device with the specific channel length can be successfully fabricated, leading to an ideal metal/semiconductor interfaces at the drain and source with less diffusion, defeats, chemical bonding and strain, as previously demonstrated using conventional electron beam lithography and high-vacuum thermal deposition [19,20]. A high-resolution cross-sectional TEM image in Figure 2b displays the interface structure of as-prepared FET device. In addition, cross-sectional TEM measurements exhibit the elemental distributions of Au, W and Se in the Au/WSe_2_ interface, and it is observed that Se does not invade the polycrystalline Au surface. It should be noted that Se and W distribution length shown is relatively larger than the real value due to the long-time integration process of images. Furthermore, FIB-STEM images of WSe_2_ on Au foil (as shown in Figure S6) confirm an intact WSe_2_ monolayer attached to the Au foil surface, indicating the uniform epitaxial growth of WSe_2_ on the gold foil surface as well as sharp and clean WSe_2_/Au interface.

### 3.3. Electrical and Optoelectronic Properties of the WSe_2_-Based Device

**Figure 3:**
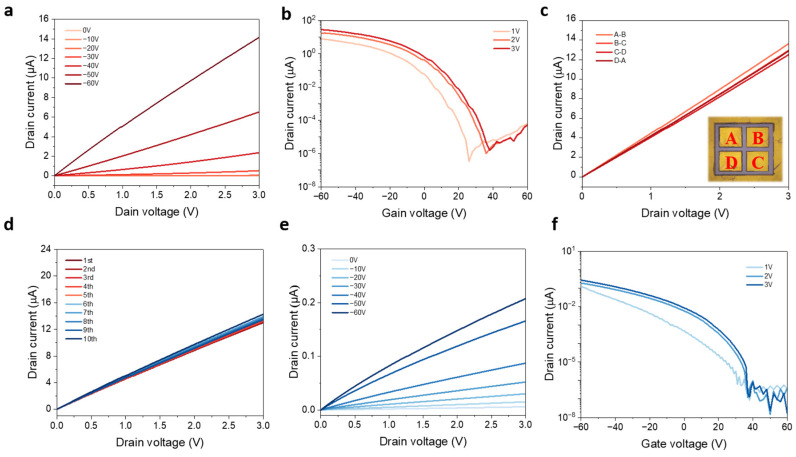
Electrical characteristics of the back-gate WSe_2_ FETs with transfer-free method and conventional thermal deposition method. (**a**) I_ds_-V_ds_ output curves and (**b**) I_ds_-V_gs_ transfer curves of monolayer WSe_2_ FETs using transfer-free method. (**c**) I_ds_-V_ds_ output curves of transfer-free devices measured between adjacent Au electrodes. (**d**) I_ds_-V_ds_ output characteristics of transfer-free devices measured within several weeks. (**e**) I_ds_-V_ds_ output curves and (**f**) I_ds_-V_gs_ transfer curves of monolayer WSe_2_ FETs with Au electrodes thermally deposited.

The electronic properties of the as-fabricated transfer-free WSe_2_ FETs are evaluated, and standard measurements are conducted under ambient conditions to obtain the device performance. Figure 3a presents the I_ds_-V_ds_ output characteristic of the device at various gate voltages, with linear curves shown suggesting that ohmic-like contacts are formed between the Au electrodes and the underneath WSe_2_. With V_ds_ bias, when the gate voltage is equal to zero (OFF working state), the current is limited by the barrier between the Au electrodes and monolayer WSe_2_. However, when a sufficient negative back-gate voltage is applied (ON working state), more holes can be transported due to the lower barrier as the Fermi level is shifted down to near the valence band of WSe_2_. As shown in Figure 3b, the I_ds_-V_g_ transfer characteristic is obtained at different drain biases from 1 to 3 V, with the back-gate voltage sweeping from −60 to 60 V. These transfer curves indicate typical p-type semiconductor characteristic of CVD-grown monolayer WSe_2_ with direct Au metal contact. In addition, when a 1 V bias is applied between drain and source, the corresponding ON/OFF ratio is about 2 × 10^6^, and on current (I_on_) it is as high as 7.84 × 10^−6^ A. The field effect mobility can be calculated using following equation:

(1) $$\mu =\left({\mathrm{dI}}_{\mathrm{ds}}/{\mathrm{dV}}_{g}\right)\times \left(L/({\mathrm{WC}}_{i}V_{\mathrm{ds}}\right)$$

where L is the channel length, W is the channel width, and $C_{i}\;$is the gate capacitance. Apart from 300 nm-thick SiO_2_ (1.15 × 10^−8^ F cm^−2^), 150 nm-thick PMMA is also taken into consideration [21] (thickness is shown in Figure S7). The final value of $C_{i}$ is calculated to be 7.35 × 10^−9^ F cm^−2^. Therefore, the mobility extracted from the transfer curve is around 62.95 cm^2^ V^−1^ s^−1^ when V_ds_ = 1 V. Additionally, devices are measured between different patterned electrodes to demonstrate the film quality, and results in Figure 3c illustrate the good uniformity of as-grown monolayer WSe_2_ film, which is ready for potential large-scale nanofabrication processes. Moreover, it is worth noting that as-fabricated transfer-free devices can maintain their original performance after weeks as no obvious device decay can be seen in Figure 3d, further suggesting the stability of our transfer-free fabrication approaches. For comparison, another batch of WSe_2_ FETs is fabricated using conventional electron beam lithography followed by high vacuum thermal deposition, resulting in undesirable output and transfer characteristics, as shown in Figure 3e,f, respectively. Not surprisingly, the evaporated contact device only exhibits mobility of 0.40 cm^2^ V^−1^ s^−1^ when V_ds_ = 1 V. According to a previous study, chemical interaction exists between thermally evaporated Au and WSe_2_ and thus hampers electrical properties, while the interlayer interaction between transferred Au electrodes and WSe_2_ is weak, barely impacting the intrinsic semiconducting behaviour [19]. Furthermore, the output current of monolayer WSe_2_ FETs with Au electrodes thermally deposited suffer significant drop after weeks (seen in Figure S8); the reason could be the poor interface contact between the Au electrodes and the channel TMD material. In our case, devices fabricated via the transfer-free method exhibit much better device performance and stability than that conducted by the conventional evaporation method due to the sharp and clean interface between electrode metal and underneath WSe_2_.

**Figure 4:**
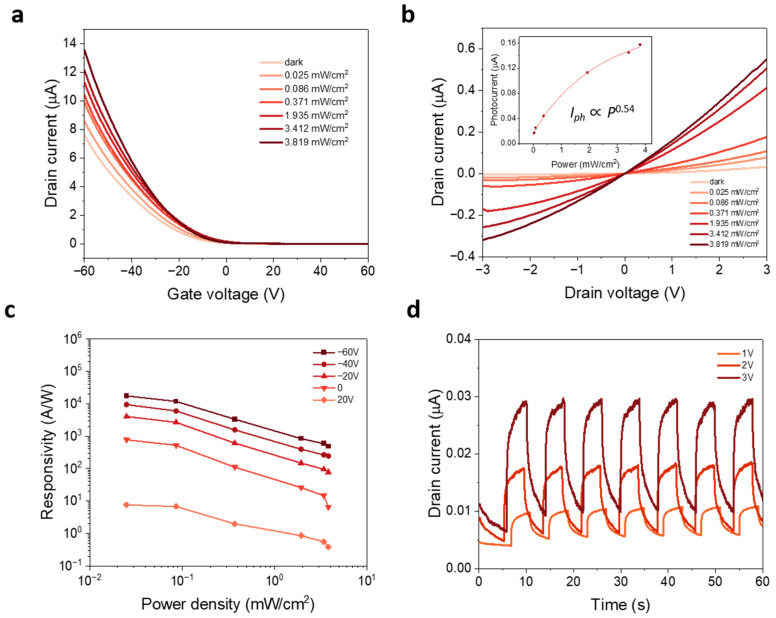
(**a**) Transfer characteristics of transfer-free monolayer WSe_2_-based photodetectors under different 532 nm laser power densities. (**b**) I_ds_-V_ds_ output curves under different light illuminations. (Insert shows the device photocurrent as a function of P). (**c**) Dependence of the responsivity on the power density with different values of back-gate voltage. (**d**) Time-dependent dynamic photoresponse at different bias voltage under 532 nm incident light illumination with a power intensity of 3.05 mW/cm^2^.

On the other hand, the photoconduction properties of WSe_2_-based monolayer were investigated by illuminating a 532 nm laser on the device channels, where the optical power P of the laser was varied. Figure 4a depicts the transfer curve of the transfer-free WSe_2_ device at V_ds_ = 1 V with different illumination power densities. Due to the photogating effect, a higher photocurrent is generated, and V_th_ is shifted to a more positive gate voltage with stronger illumination power densities. It showed that in the depletion region at the metal/TMDs interface, the depletion region due to the Schottky barrier can extend to several micrometers in the channel [22]. The ohmic-like metal contact leads to narrow depletion region where voltage drop related to adsorbates, defects or charge impurities at interfaces becomes important as the photodesorption or photoexcitation through defect or charge impurity states to band edge may contribute to photocurrents in the ON state [23]. Figure 4b shows the I_ds_-V_ds_ output characteristic of the device at various laser power densities. The relationship between $I_{\mathrm{ph}}\;$and P follows the power law dependence, ${\;I}_{\mathrm{ph}}\propto P^{\gamma }$, where exponential γ describes the dominant photocurrent generation mechanism. Here, γ is determined to be ~0.54 and this nonlinear relationship discards the photothermoelectric effect as the origin of the observed photoresponse. For WSe_2_, the nonlinear dependency has been reported to be 0.5, while for other TMDs such as MoS_2_, the value lies between 0.5 and 0.7 [24].

One of the critical figures of merit for photodetector performance is photoresponsivity R, which is defined as

(2) $$R={\;I}_{\mathrm{ph}}/{\;P}_{\mathrm{laser}}$$

${\;I}_{\mathrm{ph}}$ is photocurrent, which is defined as

(3) $${\;I}_{\mathrm{ph}}={\;I}_{\mathrm{light}}-{\;I}_{\mathrm{dark}}$$

while ${\;P}_{\mathrm{laser}}$ is the laser power. As shown in Figure 4c, when gate voltage shifts to more negative values, the corresponding responsivity values increase significantly, indicating working state switches from OFF to ON. Moreover, photoresponsivity enhancement can be observed with a lower power density, which is derived from the low density of occupied trap states in monolayer WSe_2_ or at the WSe_2_/Au interfaces under dim laser illumination [25]. The lower laser power density also results in lesser recombination rate of the photoexcited carrier, improving the photoresponsivity of the device [26]. As a result, a photoresponsivity of ~1.7 × 10^4^ A W^−1^ is achieved when the photodetector operates at V_ds_ = 1 V and V_g_ = −60 V, which is comparable to previously reported values [24,27]. The high photoresponsivity indicates the photoinduced electron-hole pairs can be efficiently separated and then transported through sharp and clean WSe_2_/Au interface to the Au electrodes. Furthermore, to determine the ability of device to detect weak optical signals, the detectivity $D^{*}$ is calculated using the formula $D^{*}={\mathrm{RA}}^{1/2}/{\left(2{e\;I}_{\mathrm{dark}}\right)}^{1/2}$, where R is responsivity, A is the effective device area, e is the elementary charge and ${\;I}_{\mathrm{dark}}$ represents the dark current. The $D^{*}$ is calculated to be ~1.2 × 10^13^ Jones for ${\;P}_{\mathrm{laser}}\;$~0.025 mW/cm^2^ at V_ds_ = 1 V. Note that this detectivity value is comparable to commercial InGaAs photodetectors (10^12^–10^13^) [28]. Figure 4d shows the time-dependent photocurrent of a transfer-free monolayer WSe_2_ device under a power density of 3.06 mW/cm^2^ at different V_ds_. The magnitude of photocurrent increases as the drain voltage rises from 1 V to 3 V. The photocurrent reaches the peak value with 532 nm incident light illumination, as shown in Figure S9a, which is measured under a power density of 3.06 mW/cm^2^ at V_ds_ = 1 V. As presented in Figure S9b, the response and recovery times are measured to be 0.7 s and 2.4 s, respectively. Generally speaking, for a photodetector, there is a trade-off between high responsivity and fast response [27]. These relatively slow responses and recovery behaviors presumably occur due to long device channel length.

## 4. Conclusions

In conclusion, we have demonstrated the growth of high-quality uniform monolayer WSe_2_ by CVD using polycrystalline Au as substrates. Spectroscopic and microscopic studies reveal the excellent crystalline quality of monolayer WSe_2_ within a centimeter-size scale, enabling the potential for large-scale optoelectronic applications. By applying a novel transfer-free fabrication method, the sharp and clean interface between monolayer WSe_2_ and Au maintains, which provides WSe_2_ FETs with much better electrical properties when compared to those with thermally deposited Au drains and sources. Moreover, the transfer-free WSe_2_-based photodetectors exhibit prominent photoresponse, with a high photoresponsivity of ~1.7 × 10^4^ A W^−1^ at V_ds_ = 1 V and V_g_= −60 V, and a maximum detectivity value of ~1.2 × 10^13^ Jones. These findings provide important information for the large-scale production of large-area and continuous films of high-quality monolayer WSe_2_ and pave the way for their applications in next-generation electronics and optoelectronics.

## Data Availability

Data are contained within the article and Supplementary Materials.

## Supplementary Materials

The following supporting information can be downloaded at: https://www.mdpi.com/article/10.3390/nano13081368/s1. Figure S1. (a) Picture of self-designed experimental schema (after growth process). (b) Pattern designed in the laser writer software for FET electrodes and optical image of WSe_2_ FETs after the etching process. Figure S2. (a) Temperature program of the CVD growth process for monolayer WSe_2_ film. (b) Optical microscopic images of monolayer WSe_2_ film growth on Au substrate. (c) AFM image collected from the as-grown monolayer WSe_2_ film on Au foil. (d) AFM image of the edge of as-transferred WSe_2_ film and the corresponding height profile acquired along the yellow section dash line. Figure S3. High-magnification optical microscopic image of the as-grown monolayer WSe_2_ film on SiO_2_/Si substrate. Figure S4. Contrast analysis results of the diffraction spots using STEM. Figure S5. The top view pictures of the sandwich structure corresponding to the different stages labeled with different colored rectangle marks. Figure S6. (a) Cross-section STEM images of as-prepared WSe_2_/Au sample. (b) STEM image of inner Au foil. Figure S7. (a) Cross-sectional TEM image and corresponding AFM picture of PMMA layer after Au etching. (b) Height of PMMA thin film via AFM. Figure S8. I_ds_-V_ds_ output characteristics of back-gate WSe_2_ FETs with conventional thermal deposition method measured within several weeks. Figure S9. (a) Time-dependent dynamic photoresponse under different wavelength incident light illumination with a power density of 3.06 mW/cm^2^ at V_ds_ = 1 V. (b) Transient response of the transfer-free monolayer WSe_2_-based photodetectors at V_ds_ = 1 V under 532 nm incident light illumination with a power intensity of 3.06 mW/cm^2^.

## References

[1] Chhowalla M., Jena D., Zhang H. (2016). Two-dimensional semiconductors for transistors. Nat. Rev. Mater..

[2] Shen P.-C., Su C., Lin Y., Chou A.-S., Cheng C.-C., Park J.-H., Chiu M.-H., Lu A.-Y., Tang H.-L., Tavakoli M.M. (2021). Ultralow contact resistance between semimetal and monolayer semiconductors. Nature.

[3] Mak K.F., Shan J. (2016). Photonics and optoelectronics of 2D semiconductor transition metal dichalcogenides. Nat. Photonics.

[4] Zhou H., Wang C., Shaw J.C., Cheng R., Chen Y., Huang X., Liu Y., Weiss N.O., Lin Z., Huang Y. (2015). Large Area Growth and Electrical Properties of p-Type WSe_2_ Atomic Layers. Nano Lett..

[5] Nipane A., Choi M.S., Sebastian P.J., Yao K., Borah A., Deshmukh P., Jung Y., Kim B., Rajendran A., Kwock K.W.C. (2021). Damage-Free Atomic Layer Etch of WSe_2_: A Platform for Fabricating Clean Two-Dimensional Devices. ACS Appl. Mater. Interfaces.

[6] Xu X., Zhang Z., Dong J., Yi D., Niu J., Wu M., Lin L., Yin R., Li M., Zhou J. (2017). Ultrafast epitaxial growth of metre-sized single-crystal graphene on industrial Cu foil. Sci. Bull..

[7] Wang L., Xu X., Zhang L., Qiao R., Wu M., Wang Z., Zhang S., Liang J., Zhang Z., Zhang Z. (2019). Epitaxial growth of a 100-square-centimetre single-crystal hexagonal boron nitride monolayer on copper. Nature.

[8] Gao Y., Hong Y.-L., Yin L.-C., Wu Z., Yang Z., Chen M.-L., Liu Z., Ma T., Sun D.-M., Ni Z. (2017). Ultrafast Growth of High-Quality Monolayer WSe_2_ on Au. Adv. Mater..

[9] Choi S.H., Kim H.J., Song B., Kim Y.I., Han G., Nguyen H.T.T., Ko H., Boandoh S., Choi J.H., Oh C.S. (2021). Epitaxial Single-Crystal Growth of Transition Metal Dichalcogenide Monolayers via the Atomic Sawtooth Au Surface. Adv. Mater..

[10] Shi J., Zhang X., Ma D., Zhu J., Zhang Y., Guo Z., Yao Y., Ji Q., Song X., Zhang Y. (2015). Substrate facet effect on the growth of monolayer MoS_2_ on Au foils. ACS Nano.

[11] Leong W.S., Wang H., Yeo J., Martin-Martinez F.J., Zubair A., Shen P.-C., Mao Y., Palacios T., Buehler M.J., Hong J.-Y. (2019). Paraffin-enabled graphene transfer. Nat. Commun..

[12] Zhang Z., Du J., Zhang D., Sun H., Yin L., Ma L., Chen J., Ma D., Cheng H.-M., Ren W. (2017). Rosin-enabled ultraclean and damage-free transfer of graphene for large-area flexible organic light-emitting diodes. Nat. Commun..

[13] Lin Y.-C., Lu C.-C., Yeh C.-H., Jin C., Suenaga K., Chiu P.-W. (2012). Graphene annealing: How clean can it be?. Nano Lett..

[14] Wang Z., Xu W., Li B., Hao Q., Wu D., Qi D., Gan H., Xie J., Hong G., Zhang W. (2022). Selective Chemical Vapor Deposition Growth of WS2/MoS_2_ Vertical and Lateral Heterostructures on Gold Foils. Nanomaterials.

[15] Wang Q.H., Kalantar-Zadeh K., Kis A., Coleman J.N., Strano M.S. (2012). Electronics and optoelectronics of two-dimensional transition metal dichalcogenides. Nat. Nanotechnol..

[16] Huang J., Yang L., Liu D., Chen J., Fu Q., Xiong Y., Lin F., Xiang B. (2015). Large-area synthesis of monolayer WSe_2_ on a SiO_2_/Si substrate and its device applications. Nanoscale.

[17] Liu B., Fathi M., Chen L., Abbas A., Ma Y., Zhou C. (2015). Chemical vapor deposition growth of monolayer WSe_2_ with tunable device characteristics and growth mechanism study. ACS Nano.

[18] Liang J., Xu K., Toncini B., Bersch B., Jariwala B., Lin Y.C., Robinson J., Fullerton-Shirey S.K. (2019). Impact of Post-Lithography Polymer Residue on the Electrical Characteristics of MoS_2_ and WSe_2_ Field Effect Transistors. Adv. Mater. Interfaces.

[19] Liu Y., Guo J., Zhu E., Liao L., Lee S.-J., Ding M., Shakir I., Gambin V., Huang Y., Duan X. (2018). Approaching the Schottky–Mott limit in van der Waals metal–semiconductor junctions. Nature.

[20] Jung Y., Choi M.S., Nipane A., Borah A., Kim B., Zangiabadi A., Taniguchi T., Watanabe K., Yoo W.J., Hone J. (2019). Transferred via contacts as a platform for ideal two-dimensional transistors. Nat. Electron..

[21] Yue L., Meng F., Ren D., Luo S. (2019). Top-gate In–Al–Zn–O thin film transistor based on organic poly(methyl methacrylate) dielectric layer. J. Mater. Sci. Mater. Electron..

[22] Zhang W., Chiu M.-H., Chen C.-H., Chen W., Li L.-J., Wee A.T.S. (2014). Role of Metal Contacts in High-Performance Phototransistors Based on WSe_2_ Monolayers. ACS Nano.

[23] Freitag M., Tsang J.C., Bol A., Yuan D., Liu J., Avouris P. (2007). Imaging of the Schottky barriers and charge depletion in carbon nanotube transistors. Nano Lett..

[24] Bandyopadhyay A.S., Adhikari N., Kaul A.B. (2019). Quantum Multibody Interactions in Halide-Assisted Vapor-Synthesized Monolayer WSe_2_ and Its Integration in a High Responsivity Photodetector with Low-Interface Trap Density. Chem. Mater..

[25] Lopez-Sanchez O., Lembke D., Kayci M., Radenovic A., Kis A. (2013). Ultrasensitive photodetectors based on monolayer MoS_2_. Nat. Nanotechnol..

[26] Wang Y., Zhang Y., Lu Y., Xu W., Mu H., Chen C., Qiao H., Song J., Li S., Sun B. (2015). Hybrid Graphene–Perovskite Phototransistors with Ultrahigh Responsivity and Gain. Adv. Opt. Mater..

[27] Xie Y., Wu E., Hu R., Qian S., Feng Z., Chen X., Zhang H., Xu L., Hu X., Liu J. (2018). Enhancing electronic and optoelectronic performances of tungsten diselenide by plasma treatment. Nanoscale.

[28] Gong X., Tong M., Xia Y., Cai W., Moon J.S., Cao Y., Yu G., Shieh C.-L., Nilsson B., Heeger A.J. (2009). High-Detectivity Polymer Photodetectors with Spectral Response from 300 nm to 1450 nm. Science.

