# Supplementary material for: Electronic and Optoelectronic Monolayer WSe2 Devices via Transfer-Free Fabrication Method
Source: Nanomaterials (Basel). 2023 Apr 14;13(8):1368. doi: 10.3390/nano13081368 (PMC10145331; doi:10.3390/nano13081368)
Supplement: Supplementary file 1 [file nanomaterials-13-01368-s001.zip › nanomaterials-2302302-supplementary.pdf]

nanomaterials

Supplementary Materials

Electronic and Optoelectronic Monolayer WSe_2 Devices via Transfer-Free Fabrication Method

Zixuan Wang [1,2], Yecheng Nie [2], Haohui Ou [2], Dao Chen [2], Yingqian Cen [2], Jidong Liu [2], Di Wu [2], Guo Hong [3,*], Benxuan Li [2,4,*], Guichuan Xing [1,*] and Wenjing Zhang [2,*]

1 Joint Key Laboratory of the Ministry of Education, Institute of Applied Physics and Materials Engineering, University of Macau, Avenida da Universidade, Taipa, Macao SAR 999078, China
2 International Collaborative Laboratory of 2D Materials for Optoelectronics Science and Technology of Ministry of Education, Institute of Microscale Optoelectronics, Shenzhen University, Shenzhen 518060, China
3 Department of Materials Science and Engineering & Center of Super-Diamond and Advanced Films, College of Engineering, City University of Hong Kong, 83 Tat Chee Avenue, Kowloon, Hong Kong SAR 999077, China
4 Electrical Engineering Division, Engineering Department, University of Cambridge, 9 JJ Thomson Avenue, Cambridge CB3 0FA, UK
* Correspondence: guohong@cityu.edu.hk (G.H.); bl398@cam.ac.uk (B.L.); gcxing@um.edu.mo (G.X.); wjzhang@szu.edu.cn (W.Z.)

Citation: Wang, Z.; Nie, Y.; Ou, H.; Chen, D.; Cen, Y.; Liu, J.; Wu, D.; Hong, G.; Li, B.; Zhang, W. Electronic and Optoelectronic Monolayer WSe2 Devices via Transfer-Free Fabrication Method. *Nanomaterials* **2023**, *13*, 1368. https://doi.org/10.3390/nano13081368

Academic Editor: Jun-ho Yum

Received: 9 March 2023
Revised: 6 April 2023
Accepted: 12 April 2023
Published: 14 April 2023

Copyright: © 2023 by the authors. Licensee MDPI, Basel, Switzerland. This article is an open access article distributed under the terms and conditions of the Creative Commons Attribution (CC BY) license (https://creativecommons.org/licenses/by/4.0/).

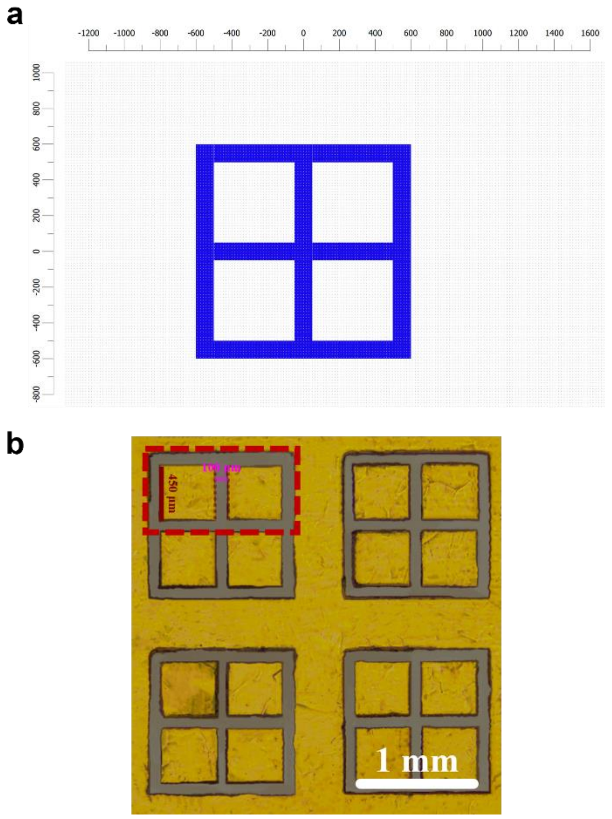

Figure S1. (a) Pattern designed in the laser writer software for FET electrodes. (b) Optical image of WSe_2 FETs after etching process.

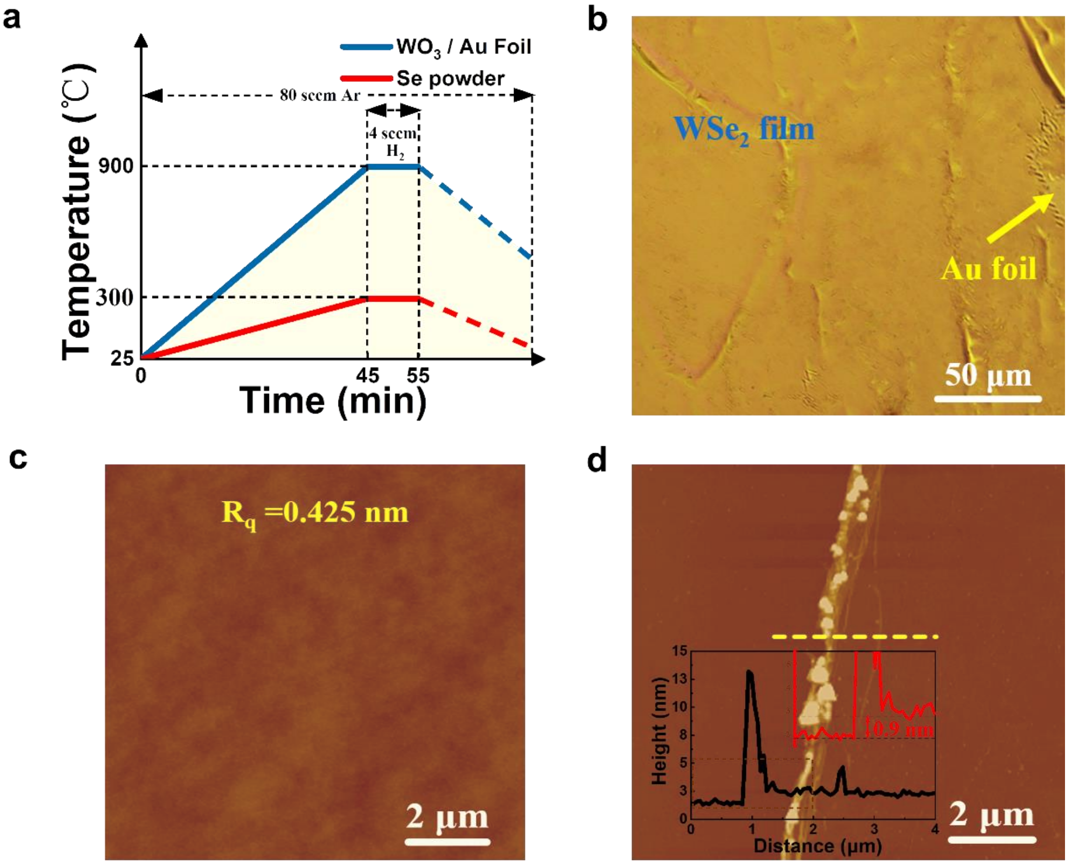

Figure S2. (a) Temperature program of the CVD growth process for monolayer WSe_2 film. (b) Optical microscopic images of monolayer WSe_2 film growth on Au substrate. (c) AFM image collected from the as-grown monolayer WSe_2 film on Au foil. (d) AFM image of the edge of as-transferred WSe_2 film and the corresponding height profile acquired along the yellow section dash line.

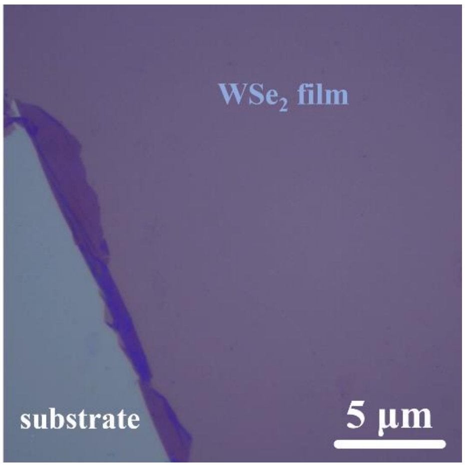

Figure S3. High-magnification optical microscopic image of the as-grown monolayer WSe_2 film on SiO_2/Si substrate.

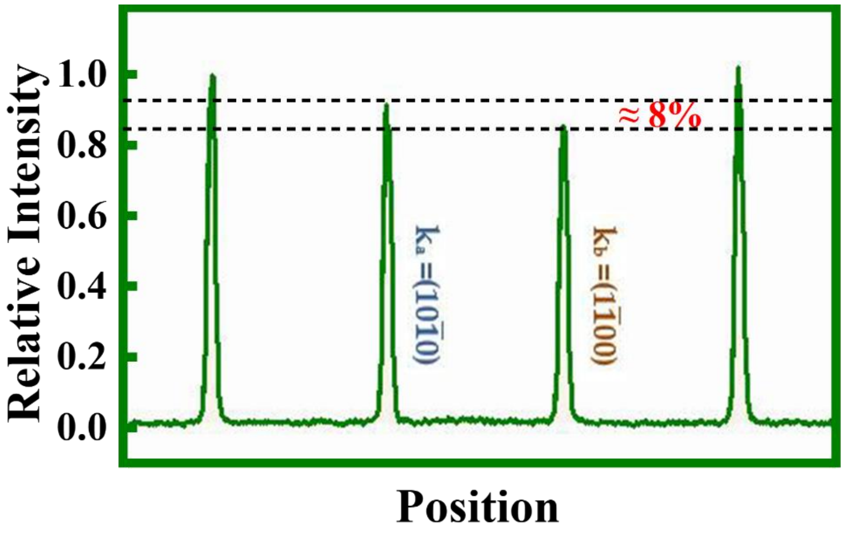

Figure S4. Contrast analysis results of the diffraction spots using STEM.

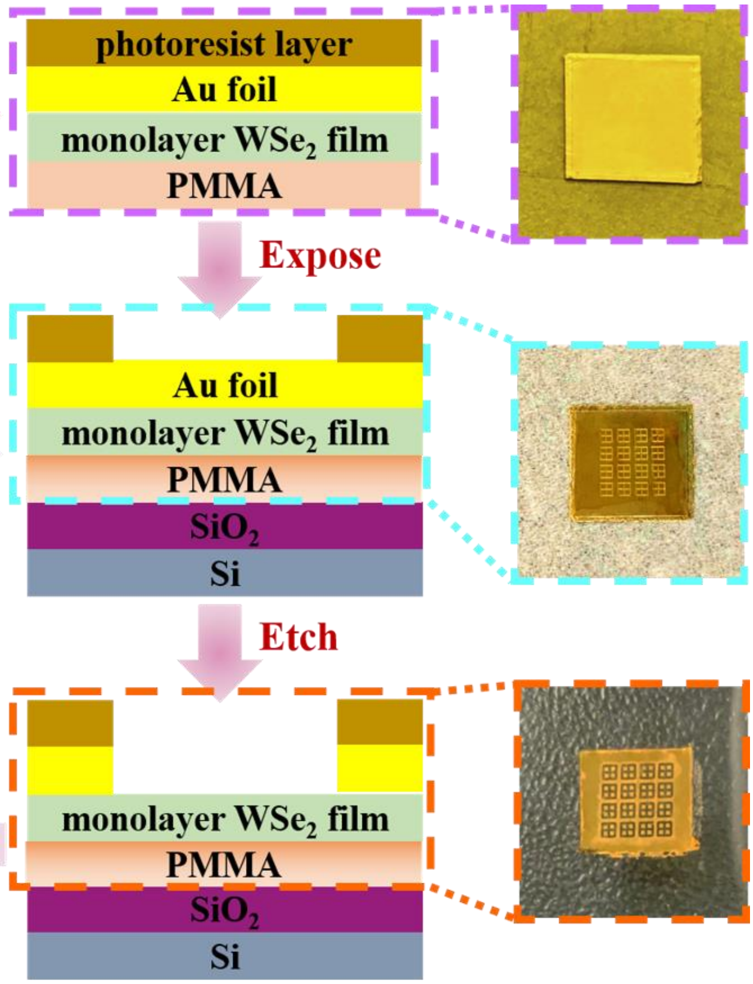

Figure S5. The top view pictures of the sandwich structure corresponding to the different stages labelled with different coloured rectangle marks.

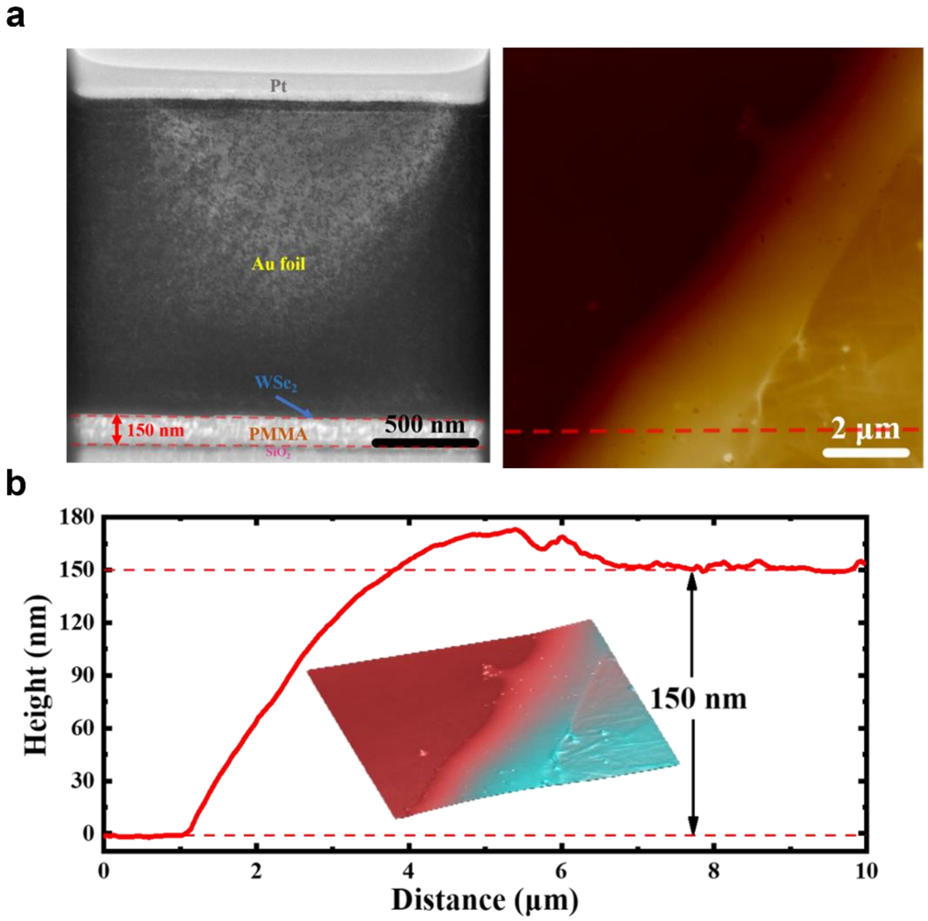

Figure S6. (a) Cross-sectional TEM image and corresponding AFM picture of PMMA layer after Au etching. (b) Height of PMMA thin film via AFM.

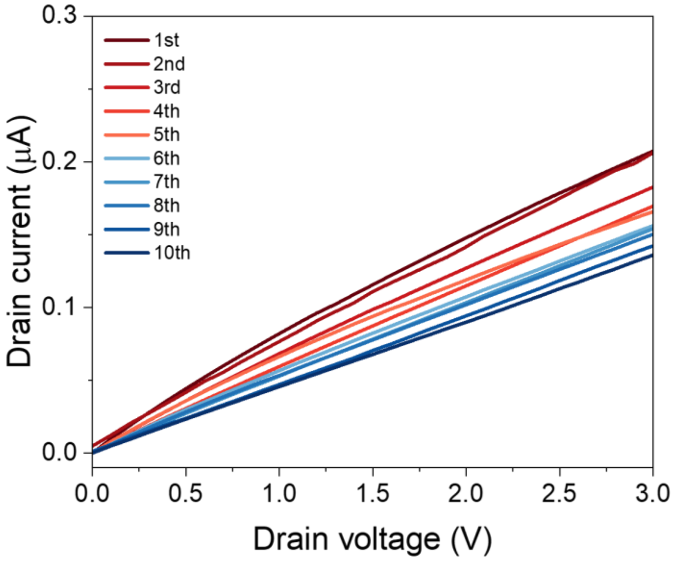

Figure S7. I_{ds}-V_{ds} output characteristics of back-gate WSe_2 FETs with conventional thermal deposition method measured within several weeks.

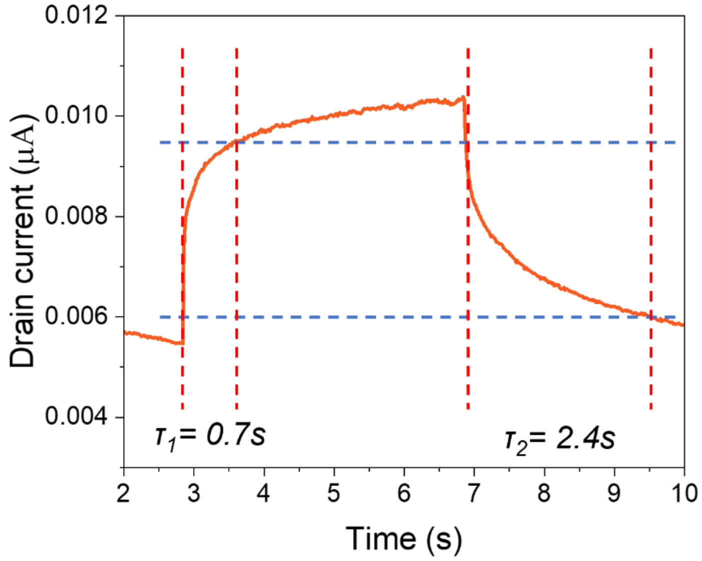

Figure S8. Transient response of the transfer-free monolayer WSe_2-based photodetectors at V_{ds}=1V under 532 nm incident light illumination with a power intensity of 3.05 mW/cm^2.

Disclaimer/Publisher's Note: The statements, opinions and data contained in all publications are solely those of the individual author(s) and contributor(s) and not of MDPI and/or the editor(s). MDPI and/or the editor(s) disclaim responsibility for any injury to people or property resulting from any ideas, methods, instructions or products referred to in the content.
